# Papillary thyroid carcinoma presenting as miliary nodules on chest roentgenogram in the paediatric setting: A case report

**DOI:** 10.1016/j.ijscr.2020.06.058

**Published:** 2020-06-13

**Authors:** Nandi Viljoen, Marc Hendricks, Eugenio Panieri, Alessandro Pietro Aldera

**Affiliations:** a Division of Anatomical Pathology, Faculty of Health Sciences, University of Cape Town and National Health Laboratory Service - Red Cross War Memorial Children’s Hospital, Cape Town, South Africa; b Haematology-Oncology Service, Department of Paediatrics and Child Health, Faculty of Health Sciences, University of Cape Town and Red Cross War Memorial Children’s Hospital, Cape Town, South Africa; c Oncology and Endocrine Surgery Unit, Division of General Surgery, Faculty of Health Sciences, University of Cape Town and Groote Schuur Hospital, Cape Town, South Africa

**Keywords:** Papillary thyroid carcinoma, Lymphangitic carcinomatosis, Endocrine pathology

## Abstract

**Introduction:**

Papillary thyroid carcinoma (PTC) is the most common carcinoma to occur in childhood with a peak incidence between 11–17 years, and typically presents with advanced locoregional disease. Pulmonary metastases are seen in up to 46% of cases and should enter the differential diagnosis of miliary nodules seen on chest roentgenogram, even in regions where tuberculosis is endemic.

**Presentation of case:**

An 11-year-old male presented with a short history of cough, shortness of breath and constitutional symptoms. Examination revealed cervical lymphadenopathy and diffuse bilateral nodular infiltrates on the chest roentgenogram. Investigation for *Mycobacterium tuberculosis* was negative and this initiated biopsy of a cervical lymph node. Histopathological examination revealed metastatic PTC. Ultrasonography and magnetic resonance imaging (MRI) were performed for preoperative staging. The patient subsequently underwent total thyroidectomy with selective neck dissection.

**Discussion:**

There are several potential causes when dealing with miliary nodules on chest roentgenogram. Thorough interrogation of the clinical, radiological, pathological and microbiological data is required to arrive at the correct diagnosis. Postoperative adjuvant therapy with radioactive iodine is recommended in children with metastatic disease, but this should be restricted preferably to a single dose to avoid the complication of pulmonary fibrosis.

**Conclusion:**

This case highlights the differential diagnostic considerations of a patient presenting with constitutional symptoms and a miliary pattern on chest roentgenogram. Carcinomas are uncommon in children but should not be forgotten.

## Highlights

• Papillary thyroid carcinoma (PTC) is the most common carcinoma encountered in the paediatric setting.
• PTC often presents with advanced locoregional disease in children and pulmonary metastasis are seen in up to 46% of cases.
• Miliary nodules on chest roentgenogram may be caused by miliary tuberculosis but other causes including metastatic carcinoma should be considered.

## 1 Introduction

Carcinomas are uncommon in the pediatric setting, and seldom enter the differential diagnosis of a metastatic lesion. Papillary thyroid carcinoma (PTC) is the most common carcinoma encountered in children and represents up to 3% of all paediatric malignancies [1]. Miliary nodules on chest roentgenogram have a wide differential diagnosis which includes infectious, auto-immune and neoplastic causes [2]. In geographic regions where tuberculosis is endemic, this is often the leading diagnostic consideration.

This case highlights the importance of considering a differential diagnosis when faced with miliary nodules on a chest roentgenogram. Although uncommon, metastatic carcinoma should not be forgotten in the paediatric age group.

This case has been reported in line with the SCARE criteria [3].

### 1.1 Case presentation

**Fig. 1:**
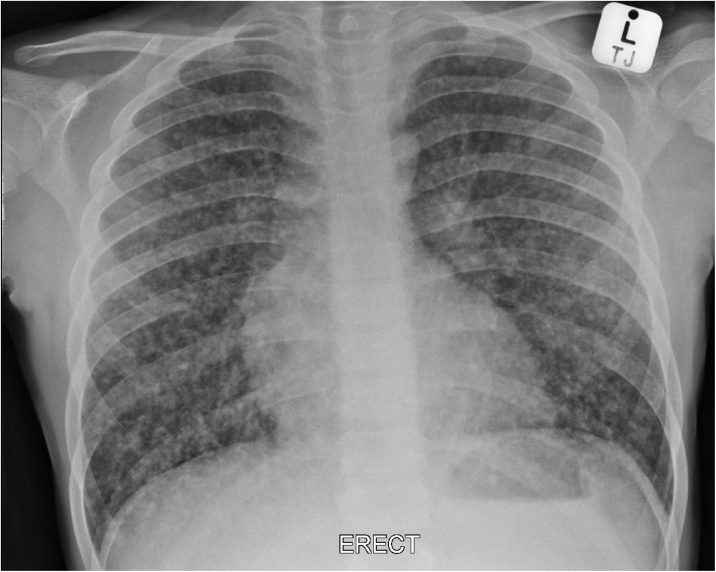
Chest roentgenogram shows bilateral diffuse micronodular opacities.

An 11-year-old male presented with worsening respiratory symptoms for two weeks, weight loss, and cervical lymphadenopathy. His past medical history included asthma and severe atopic dermatitis, which at presentation were inadequately controlled. Clinical examination revealed hypoxaemia (oxygen saturation <92% in room air), respiratory distress and enlarged non-tender cervical lymphadenopathy. The chest roentgenogram showed bilateral diffuse micronodular opacities (Fig. 1). The leading diagnostic consideration at this point was miliary tuberculosis given the high prevalence of tuberculosis in our region. However, thorough microbiological investigation, including mycobacterial culture and polymerase chain reaction (PCR) failed to demonstrate *Mycobacterium tuberculosis*.

**Fig. 2:**
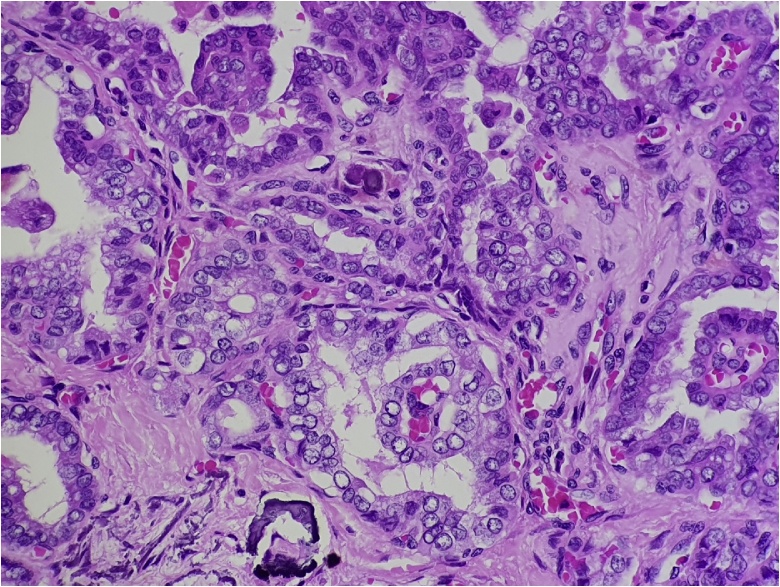
Histopathology shows malignant glands and papillary structures with psammoma bodies and cells with classic papillary thyroid carcinoma nuclear features (H&E, 400× magnification).

**Fig. 3:**
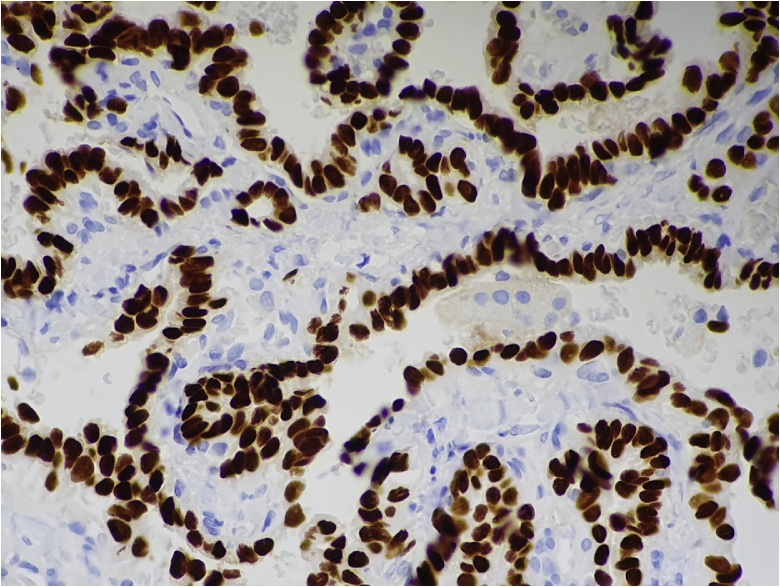
TTF1 immunohistochemistry labels the neoplastic cells (400× magnification).

Tissue was obtained from one of the enlarged cervical lymph nodes to investigate the possibility of lymphoma. Histopathology of the core biopsy showed focal involvement by metastatic carcinoma demonstrating papillary-cystic growth with scattered solid islands and psammoma bodies (Fig. 2). The neoplastic cells displayed nuclear enlargement with irregular nuclear membranes, nuclear overlap and “Orphan Annie eye” nuclear inclusions. Immunohistochemical interrogation revealed positivity with cytokeratin (CK) 7, thyroid transcription factor 1 (Fig. 3), and thyroglobulin. These findings supported the pathological diagnosis of metastatic PTC.

Retrospectively, further clinical examination revealed an ill-defined 2,5 × 1,5 cm left thyroid nodule. Ultrasonography confirmed a heterogeneous left thyroid nodule with internal calcifications. Considering the histological diagnosis, the chest roentgenogram findings were reviewed to now include carcinomatosis of the lung. Uncontrasted magnetic resonance imaging (MRI) of the mediastinum and neck revealed a lobulated, predominantly solid thyroid mass that returned heterogeneous T2 high signal and T1 low signal, with associated extensive bilateral cervical lymphadenopathy. Preoperative thyroid scintigraphy was not performed due to local unavailability of radioactive iodine during the Covid19 pandemic. Total thyroidectomy with central and modified left cervical lymph node dissection was performed, preserving the sternocleidomastoid muscle, accessory nerve and internal jugular vein, and removing nodes from zones 2–6. Surgical findings revealed a firm, ill-defined mass involving the left lobe of the thyroid, invading the strap muscles. Multiple pathological lymph nodes were noted in both the central and the left lateral compartments of the neck. Pathological evaluation of the resection specimen revealed extensive multifocal involvement by PTC, with extrathyroidal extension and widespread involvement of cervical lymph nodes. Seven out of 9 nodes in the central compartment were positive, and 15 out of 59 nodes were positive in the lateral compartment. Immunohistochemistry for BRAFV600E was negative.

Postoperative calcium-phosphate metabolism was normal, and the patient did not experience any further adverse events. The postoperative thyroid hormone status was as follows: thyroid stimulating hormone (TSH) 16.47 mU/L (normal range 0.57–4.03), thyroxine (T4) 9.0 pmol/L (8.5–13.6), thyroglobulin 4081.0 ug/L (normal range 3.5–77.0) and antithyroglobulin 15 (normal <115 U/mL). Postoperative levothyroxine was initiated because of the hiatus in receiving radioactive iodine therapy for the metastatic lung disease. Prior to initiation of thyroid hormone replacement, a whole-body diagnostic I-131 scan revealed extensive uptake of iodine in the lungs bilaterally. This finding confirmed the preoperative impression of widespread pulmonary metastases. The patient was discharged from hospital pending the first course of therapeutic radioactive iodine (RAI) to treat the metastatic lung disease.

## 2 Discussion

PTC is the most common histologic type of thyroid carcinoma in the paediatric age group and represents 85–95% of cases [1]. The incidence increases with age, with a peak in the paediatric population between the ages of 11 and 17 years [4]. Affected children are commonly euthyroid and present with an enlarging neck mass [5]. PTC in children tends to present with more aggressive disease including a higher rate of extrathyroidal extension, regional lymph node involvement and distant metastases. Pulmonary metastatic disease is frequently present and has been reported in up to 42% of cases [6,7]. The most important risk factor for developing thyroid carcinoma is previous diagnostic, therapeutic, or nuclear radiation exposure. Mutations in *BRAF* are important, particularly in cases with no prior radiation exposure [8]. The case that we present had no radiation exposure and immunohistochemistry for BRAFV600E was negative.

Sharma has recently outlined an approach to the differential diagnosis of miliary nodules on chest roentgenogram [2]. In areas of endemic tuberculosis, this is the most likely consideration and missing or delaying the diagnosis of miliary tuberculosis could prove rapidly fatal. Miliary tuberculosis typically shows micronodules on imaging which measure 1–3 mm in diameter. Another radiological clue to the diagnosis of tuberculosis is the presence of hilar lymphadenopathy. Other infectious aetiologies include histoplasmosis, mycoplasma, nocardia and blastomycosis [9]. Sarcoidosis, hypersensitivity pneumonitis, and pneumoconiosis may also present with this pattern. Airway spread from bronchogenic carcinoma can also show a miliary pattern. Haematogenous dissemination of carcinoma, particularly originating from the thyroid or kidney are other causes that are more frequently encountered in the adult population. The nodules in metastatic carcinoma are typically larger than 3 mm and hilar lymphadenopathy is not a usual feature.

The use of RAI therapy for regional and distant metastases is effective in the paediatric age group and partial or complete response is often achieved. The evidence for the routine use of therapeutic postoperative RAI in children without metastatic disease is less clear. It is critical to quantify the risk-benefit ratio as accurately as possible as there are several well-recognised toxicities. These include development of second primary malignancies, adverse reproductive effects, pulmonary fibrosis and effects on salivary glands. Marti et al. examined the development of secondary primary malignancies in a cohort of 3850 young patients who received therapeutic RAI [10]. They identified salivary gland carcinoma, leukaemia and renal carcinoma as the most common secondary primary malignancies. Potential adverse reproductive effects of RAI therapy include an increased rate of miscarriage in young women and short-term infertility in males [11,12]. These effects appear to be reversible and there is no increased risk of teratogenesis [13]. Pulmonary fibrosis is of particular concern in patients who have diffuse pulmonary metastases. Fibrosis should, however, not be induced if the current recommended guidelines for dosage are adhered to [14]. Despite incomplete remission following a single course of therapeutic RAI, declining thyroglobulin and clinically stable partial remissions can be achieved in children, and additional courses of RAI should be handled restrictively [15].

This case highlights the importance of obtaining microbiological confirmation of *Mycobacterium tuberculosis* prior to initiating therapy and discounting the possibility of other causes, and the differential diagnostic considerations of a patient presenting with constitutional symptoms and a miliary pattern on chest roentgenogram.

## Funding

None.

## Ethical approval

This is a case report and approval from an ethics body is not required.

Written informed consent has been obtained form the patient’s legal guardian.

## Consent

Written informed consent has been obtained from the patient’s legal guardian.

## Author contribution

Aldera – concept, design, writing the paper.

Viljoen – data collection, writing the paper.

Panieri – writing the paper.

Hendricks – writing the paper.

## Registration of research studies

Not applicable.

## Guarantor

Alessandro Pietro Aldera.

## Provenance and peer review

Not commissioned, externally peer-reviewed.

## Declaration of Competing Interest

There authors declare no conflict of interest.
